# Living with moderate to severe renal failure from the perspective of patients

**DOI:** 10.1186/s12882-016-0263-1

**Published:** 2016-05-16

**Authors:** K. Schipper, W. E. van der Borg, J. de Jong-Camerik, T. A. Abma

**Affiliations:** Department of Medical Humanities/EMGO+, VU University Medical Center, Post box 7057, 1081 BT Amsterdam, The Netherlands

**Keywords:** Chronic Kidney Disease, GFR, Moderate, Patient perspectives, Quality of life, Severe

## Abstract

**Background:**

Within healthcare, almost no attention is given to patients with moderate-to- severe chronic kidney disease, having a with GFR between 20 and 45 while the presumption exists that these patients already experience several problems in their lives during the course of their illness.

**Methods:**

A team of academic researchers and a renal patient participated in a qualitative study. Individual interviews (*n* = 31) and focus groups (10 participants in total) with patients having moderate-to-severe chronic kidney disease were conducted to gain insight into their everyday problems.

**Results:**

Participants mentioned several experiences that can be divided into physical, social, societal and psychological aspects as well as aspects related to healthcare. The most important findings, following under each of these categories are: 1) the experience of fatigue (physical aspects) 2) the search for acknowledgment of complaints/not enough attention given to complaints leading to overcompensation and secrecy (societal aspects) 3) work problems (societal aspects) and 4) the wish to control the disease but not receiving enough support for this (healthcare). Patients feel in general that healthcare professionals do not take them seriously in their complaints and problems.

**Conclusions:**

This study offers important new insights into an expanding group of patients having moderate-to-severe chronic kidney disease. Healthcare professionals should acknowledge their problems instead of ignoring or rejecting them and they should support patients in finding a way to deal with them. The assumptions of Personalised Care Planning could be used to support patients.

## Background

Chronic Kidney Disease (CKD), defined by reduced Glomerular Filtration Rate (GFR) and/or increased urinary albumin excretion [1] is a problem with an increasing incidence and prevalence and high costs (http://www.kidney.org, http://ekha.eu/) [2]. The prevalence is estimated to be 8–16 % worldwide [1]. One potential outcome of CKD is End-Stage Renal Disease (ESRD), requiring renal replacement therapy like dialysis or transplantation.

The Dutch Kidney Patient Association (NVN) has heard from their members that professionals in the Netherlands adhere to the assumption that complaints related to CKD only arise if GFR is lower than 20 or 30 and when a renal replacement therapy is about to start (pre-dialysis stage). This is also stated in patient education materials for instance from the Dutch Kidney Foundation (www.nierstichting.nl). A search on the Internet shows not only a Dutch tendency but also a more widespread tendency to adhere to the assumption that complaints only arise if the GFR drops below 30. Medscape, a Health Professional Network that offers specialists, primary care physicians and other health professionals and the Internet’s most robust and integrated medical information and educational tools, for instance mentions that *‘Patients with CKD stages 1–3 (GFR >30 mL/min/1.73 m*^*2*^*) are generally asymptomatic’* (http://emedicine.medscape.com/article/238798-overview).

Based on input from their members, the patient association became aware that this assumption might be incorrect and that CKD patients can experience complaints much earlier. This is also suggested by the literature; high mortality rates and prevalence of comorbid conditions, even in patients at earlier stages of CKD have been reported [3]. Members of the association requested attention for patients with moderate-to-severe CKD (GFR 20–45), their Quality of Life (QoL) and their experienced problems since they felt neglected by the association and professionals.

The existing literature consists of very few studies on the experiences of patients with moderate to severe CKD (stages 1–3) and their QoL. Most of the studies focus on patients with advanced CKD (stages 4 and 5) [3]. Based on those studies, we know that the Health Related Quality of Life (HRQOL) in patients with advanced CKD (stage 4 and 5) is significantly impaired [3]. The studies conducted on the QoL of patients in stages 1–3 describe a decreased HRQOL among patients with mild-to-moderate renal dysfunction compared with the population having normal renal function [3].

The QoL of patients with CKD is thus lower than is the QoL of people without CKD. One may expect that the QoL of patients decreases with a decreasing GFR but studies on this relationship give conflicting results. Lee and colleagues included 5555 patients with CKD stages 1–3 in a study. They described a relationship between QoL and GFR: a lower GFR is related to a lower QoL [3]. Lemos and colleagues have however described other results. They investigated 170 patients with CKD at stages 1–5 (not on dialysis yet) and concluded that the degree of renal function did not influence QoL [4]. Gender, age and family income were, however, described as affecting QoL [4].

The quantitative literature does teach us something about the QoL of patients with CKD. In these studies, QoL seems to be impaired in all stages of the disease and it is unclear whether or not there is a strong relation between GFR and QoL [3, 4]. Gender, age and family income seems to be important factors that influence QoL [4]. These insights are important but do not give us insight into the profound experiences of patients living with CKD and the factors that may influence their QoL. Qualitative studies can be helpful for a more profound understanding [5]. The literature describes several qualitative studies, for instance, on experiences with dialysis, experiences with living-related transplantation and living with a kidney transplant. However, studies on the impact of living with less advanced CKD and how patients experience their QoL and what they need in order to stay physically and mentally as healthy as possible, are lacking. Insight into patients’ experiences, coping, QoL and needs is important to fine-tune their care and to provide the necessary support. We, therefore, performed a qualitative study aimed at describing the experiences and needs of patients with moderate-to-severe kidney damage (GFR 20–45).

## Methods

### Participant selection

Patients were eligible to participate if they had a GFR between 20 and 45 for at least 3 months, were not yet at the pre-dialysis/pre-transplantation stage and were between 18 and 80 years old. The inclusion criterion of a GFR between 20 and 45 was strongly recommended by the scientific advice committee of the funder, the Dutch Kidney Foundation. The members of the committee (nephrologists) asked us to combine stage 2, 3 and 4 in order to get a more similar group with less diverging experiences.

The NVN, hospitals and the Facebook page for the Dutch Kidney Foundation were used to recruit participants. The sampling for our method of recruitment in this study was purposeful as we were selecting a rich variety of patients in terms of age, gender, GFR, hospital in which patients were treated, method of recruitment in this study (patient association, Facebook, hospitals) [6]. Patients were asked to report their latest GFR as measured by their medical doctor to check whether they actually met inclusion criteria. All patients that were directly asked by the NVN and professionals to participate were willing to participate in an interview or focus group. The research team, taking the criteria into account, made the selection of participants from those willing to participate.

### Data collection and data analysis

A team consisting of academic researchers and a renal patient conducted the study.

Actively including patients as partners in research has several advantages such as establishing trust and openness [7–13].

In the first part of the study, 31 semi-structured interviews were completed by one of the academic researchers (psychologists and anthropologist) and the patient researcher [11].

The academic researchers previously participated in courses on how to conduct qualitative studies and all interviewers were experienced in conducting qualitative studies. They took general quality criteria for the semi-structured interviews into account, such as asking open-ended questions, using different probes and avoiding jargon [14]. The interviews were aimed at obtaining insight into the experiences of patients. Participants’ stories led the interviews and a list of topics (Appendix 1) was used to check whether all topics were discussed. The interviews lasted between 60 and 120 min and were, after respondents’ verbal consent, audio-recorded and transcribed verbatim.

All interview transcripts were subjected to a thematic analysis [15]. First, all of the entire transcripts were read line by line and emerging themes were coded. New themes in the transcripts were added to the list of codes and then used to re-analyse the interviews that had already been analysed. A mix of computerised (MAXQDA) and manual techniques was used to facilitate data analysis. To enhance credibility, we adhered to the following procedures: 1) two researchers separately coded all interviews (investigator triangulation) [16], 2) all individual analyses were compared and discussed until consensus was reached [17] and 3) participants received an interpretation of the interview and were asked whether they recognised the analysis (member-check) [17]. All patients agreed. The themes of all interviews were grouped and further explored in two focus groups with other participants (2 × *n* = 5, in total *n* = 10). Focus groups can be used to deepen, explain or check data. The entire research team prepared the focus groups and a senior researcher (KS) led them, using a protocol (see Appendix 2).

After three interviews, it became clear that fatigue seemed to be an important complaint of the participants. The interviews were not analysed yet, but fatigue was a prominent theme in the three interviews. To gain better insight into the fatigue experienced, we decided to add the Checklist Individual Strength to measure the experienced fatigue [18]. All patients, including the first three patients, were asked to fill out the questionnaire after the interview or focus group. In all, 32 patients returned the questionnaire (response rate 78 %).

### Ethical considerations

The Medical Ethics Committee approved this study (including the questionnaire), and the procedures followed were in accordance with the ethical standards of the Medical Ethics Committee of the VU Medical Center. All participants voluntarily took part and none of them declined our request to participate. All participants gave verbal informed consent. Confidentiality was maintained by restricted, secure access to the data, destruction of audiotapes following transcription and de-identifying the transcripts.

## Results

In total, 41 patients participated in this study. An overview of the participants’ characteristics in the interviews and focus groups are presented in Tables 1, 2 and 3.

**Table 1:** Characteristics patients interviews (*n* = 31)

Respondent	Gender	Age range	Stage	CIS fatigue
R1	F	36–45	3b	-
R2	F	46–55	4	Severe
R3	M	46–55	3b	-
R4	F	56–65	4	Normal
R5	F	26–35	4	Severe
R6	F	26–35	3a	Severe
R7	F	56–65	4	Severe
R8	F	36–45	4	Severe
R9	F	46–55	4	Severe
R10	F	56–65	3b	Severe
R11	F	18–25	3b	Severe
R12	M	36–45	4	Severe
R13	M	26–35	4	Severe
R14	F	46–55	4	Severe
R15	M	26–35	4	Severe
R16	M	36–45	4	Severe
R17	M	66–75	3b	Normal
R18	M	56–65	3b	-
R19	F	46–55	4	-
R20	F	36–45	3b	Severe
R21	M	46–55	4	-
R22	F	36–45	3b	-
R23	F	36–45	4	-
R24	F	46–55	4	-
R25	M	46–55	3a	-
R26	M	18–25	3b	Severe
R27	F	46–55	3b	Severe
R28	M	26–35	3b	Severe
R29	F	56–65	3b	-
R30	F	56–65	4	-
R31	M	56–65	4	-

Stage 3A: GFR between 45 and 59, stage 3B: GFR between 30 and 44 and stage 4: GFR 15–29 (in our study no GFR’s lower than 20)

**Table 2:** Characteristics patients focus groups (*n* = 10)

Respondent	Gender	Age range	Stage	CIS fatigue
R32	M	46–55	3a	Severe
R33	F	56–65	3b	Normal
R34	M	56–65	3a	Severe
R35	F	56–65	4	Normal
R36	M	56–65	3b	Normal
R37	M	66–75	3b	Normal
R38	M	36–45	4	Severe
R39	F	36–45	3b	Severe
R40	F	56–65	3b	Severe
R41	F	46–55	4	Severe

Stage 3A: GFR between 45 and 59, stage 3B: GFR between 30 and 44 and stage 4: GFR 15–29 (in our study no GFR’s lower than 20)

**Table 3:** Characteristics patients interviews and focus groups (*n* = 41)

	Percentage
Age 18–25	4,9 %
Age 26–35	12,2 %
Age 36–45	24,4 %
Age 46–55	24,4 %
Age 56–65	29,2 %
Age 66–75	4,9 %
Women	59 %
Men	41 %
GFR 20–29	49 %
GFR 30–45	51 %
Stage 3A	9,8 %
Stage 3B	41,5 %
Stage 4 (not lower than GRF 20)	48,7 %
Score severe fatigue on CIS-fatigue	75 %
Score severe fatigue on CIS-fatigue with GFR 20–29 (16 persons had a GFR between 20 and 29 and filled out the questionnaire)	(75 %)
Score severe fatigue on CIS-fatigue with GFR 30–45 (16 persons had a GFR between 30 and 45 and filled out the questionnaire)	75 %

The interviews and focus groups resulted in an overview of experiences. Participants mentioned several experiences that can be divided into physical, social, societal and psychological aspects as well as aspects related to healthcare. An overview of the aspects and its subthemes is given in Table 4. All of these themes are described below. The findings are illustrated, using the participants’ quotes. Each quotation is marked with the number of the participant as well as their gender, age range and GFR.

**Table 4:** Overview of themes and subthemes

Theme	Subthemes
Physical aspects	-
Social aspects	Growing up
	Intimate relationships and sexuality
	Desire to have a child
	Family life and raising children
	Social contacts
Societal aspects	Ignorance and image formation
	Trivialising
	Pressure to legitimate
	Fear of stigmatisation and prejudice
	Embarrassment and identity
	Education, work and social security
Healthcare	Education
	Contact with professionals
	Multidisciplinary care and self-management
	Experimental care and future treatment
Psychological aspects	Deterioration - insecurity and trust
	Coping

### Physical aspects

A few participants did not experience any physical problems, but the majority of the participants mentioned several complaints. Problems expressed were fatigue, concentration and memory problems, cramp, being unfit, feeling cold, gout, restless legs, kidney pain and itching. Fatigue was mentioned as the most problematic complaint:

*‘My battery is full when I wake up in the morning, but my battery runs low during the day’. (R6, F, 26–35 years, GFR 45)*

*‘I can’t describe it. How to describe fatigue? For me it is having sudden moments without having any energy and without being able to do anything’. (R4, F, 56–65 years, GFR 23)*

This qualitative finding was validated by the CIS-Fatigue questionnaire. A total of 75 % of the participants (including those with lower and those with higher GFR) who filled out the questionnaire experienced severe fatigue.

The accumulation of physical problems was expressed as a *‘never-ending story’,* which was hard to deal with:

*‘The most difficult part of having CKD is the fact that it never stops. It is a never-ending story and you are running from problem to problem. It’s an accumulation’. (R14, F, 46–55 years, GFR 20)*

Dealing with the problems was especially difficult because participants did not know whether to attribute the complaints to the disease or to isolated problems:

*‘Is it a direct consequence of your disease or is it the result of other circumstances? It’s hard to make that distinction’. (R32, M, 46–55 years, GFR 45)*

### Social aspects

#### Growing up

Having CKD as a child influenced the dynamics of childhood and adolescence. Participants had to make other choices and were not able to follow the same route as their peers. They felt that they were treated as being different and that the environment usually did not adjust to their illness. Their possibilities and capacities declined because of the illness, while the possibilities of their peers increased:

*‘I had to make other choices, I wasn’t able to follow their [peers] paths’. (R38, M, 36–45 years, GFR 27)*

#### Intimate relationships and sexuality

The illness may influence the intimate relationships of patients. Lack of communication was mentioned as one of the causes of relational problems and in some cases, CKD led to divorce. Finding new intimate relationships was also described as difficult. Participants mentioned their doubts about the question ‘what to tell and what not to tell?’ They were afraid of losing their potential partner due to the disease. Some participants actually experienced people being scared off by the disease:

*‘It was scary to tell my current boyfriend about the disease. I was afraid that he would leave me’. (R20, F, 36–45 years, GFR 42)*

Fears for the future were also expressed. Participants feared for the continuation and endurance of their intimate relationship in the light of further progression of the disease:

*‘I can be afraid if I think about the future … Will he still love me if I have more restrictions? And can we stay partners on equal terms?’ (R20, F, 36–45 years, GFR 42)*

Related to that is the fact that participants mentioned changes in their sexual functioning such as decreased libido and as a result of this, for some men, there were feelings of ‘being less masculine’:

*‘You are not a real man anymore because of your decreased libido. It feels as if I have failed’. (R16, M, 36–45 years, GFR 25)*

#### Desire to have a child

Having CKD influenced some participants’ decisions as to whether they did or did not want to have children. The insecure future, the possible harmful effect of being pregnant on kidney functioning, and heredity were taken into account:

*‘The nephrologist has said that I can have children. But he also mentioned the risks for my kidney. So I was thinking … We should not do it … And what can I offer a child if I’m in hospital three times a week for dialysis’. (R23, F, 36–45 years, GFR 35)*

Some participants felt they had to defend their choices:

*‘It’s hard to defend yourself. People are always asking why we don’t have children. They have their own opinions and prejudices. The worst are those people who say “that the disease isn’t that bad and that you never know how medical science will develop”. Of course they are right but it is my choice and I don’t want my children to have an ill mother’. (R20, F, 36–45 years, GFR 42)*

Some female participants, however, did not have any choice at all. They were not able to have children due to their CKD. Other participants had difficulty becoming pregnant and needed fertility treatment.

#### Family life and raising children

For those that did have children raising them may be negatively influenced by CKD. Participants mentioned feelings of being restricted in regard to family activities, which led to feelings of sadness or guilt:

*‘You don’t live the life you would like to live. I can’t lead the life I envisioned for myself and my kids. … I’m just trying to survive’. (R22, F, 36–45 years, GFR 35)*

Furthermore, they did not like the fact that their children had to deal with the consequences of their disease. They found it important that their children should be allowed to be children, despite the disease of one of their parents. However, this ideal turned out to be difficult or sometimes impossible. In some cases, this gave rise to tensions between parents since their children expressed their dissatisfaction to the healthy parent.

Finally, participants expressed concerns about the feelings of the healthy partner (is he/she able to deal with my disease?) and possible heredity (do my children have the same disease or not?):

*‘Ï’m worrying about my wife since she has to deal with my disease and the worries about the potential disease of our children’. (R18, M, 56–65 years, GFR 39)*

#### Social contacts

CKD influenced social contacts, often in a negative way. Friendships were lost, because participants did not have enough energy to be an active partner in friendships or to be involved in activities. Work was stressed as being important for social contacts:

*‘Conversations at parties stagnate when you say that you don’t work’. (R10, F, 56–65 years, GFR 38)*

Losing work due to CKD often resulted in losing contacts. Some participants were afraid of losing friends in advance, but the reality was not as bad as they had anticipated. Other participants explained how they consciously chose to maintain fewer friendships. Having fewer friends made it easier to keep in touch with those contacts:

*‘I used to have many friends and my network was getting bigger and bigger. And I assumed that I needed to maintain all these contacts. But I’m no longer able to do that so I had to make choices: who is really important to me and who is not? Who are the people I want to give my time and energy to and who are not’? (R6, F, 26–35 years, GFR 45)*

### Societal aspects

#### Ignorance and image formation

Many participants expressed their struggle with existing ideas about CKD in society. Those around the interviewees were often perceived as associating CKD with dialysis or transplantation. Participants often perceived those in their environment as being unaware of the problems of this particular category of patients:

*‘People think about dialysis or transplantation when I say that I’m a renal patient. They don’t know the other options. It is not seen as renal disease if you are not on dialysis or if you don’t have a donor kidney’. (R9, F, 46–55 years, GFR 26)*

#### Trivialising

Some participants do not feel that their complaints are taken seriously, in part due to inadequate image formation: ‘*it cannot be that bad if you are not dialysing yet’.*

The participants themselves, on the other hand, also tend to trivialise their disease and complaints by comparing their disease with more severe diseases, such as cancer, although the prospects of many forms of cancer are better compared to CKD:

*‘I don’t want to bother other people. It would be different if you had cancer’. (R37, M, 66–75 years, GFR 30)*

#### Pressure to legitimate

Participants often do not feel taken seriously by professionals. They were lacking the acknowledgement of professionals:

‘*I don’t tell my nephrologist my problems any more. She will not take them seriously*’. *(R41, F, 46–55 years, GFR 20)*

In part due to this attitude of professionals, invisibility, unfamiliarity and trivialising, participants mentioned pressure to defend themselves and their complaints. Sometimes, they felt they had to persuade others that their complaints were real, not the same as those experienced by others (everyone is fatigued sometimes) and not faked. Patients and the people in their environment struggled with the legitimacy of the disease.

*‘People don’t realise the impact of having this disease. They tend to ignore it and I think they see me as someone who fakes it’. (R22, F, 36–45 years, GFR 35)*

*‘I felt guilty and I felt that I had to defend myself constantly towards others’. (R8, F, 36–45 years, GFR 23)*

#### Fear of stigmatisation and prejudice

Some participants lived a life full of secrets: they did not want those in their environment to know about their disease because they worried about the consequences, such as losing their job:

*‘My colleagues and employer don’t know that I have CKD. I’m afraid they will use it against me’. (R20, F, 36–45 years, GFR 42)*

Participants also expressed fear of being stigmatised, facing prejudice or of no longer being taken seriously:

*‘I don’t want to have the “stamp” patient, because I don’t feel like a patient right now’. (R32, M, 46–55 years, GFR 45)*

#### Embarrassment and identity

Some participants expressed embarrassment because of their disease, their restrictions or required devices, especially those with physical restrictions. The disease and losses also negatively influenced their identity. Some participants mentioned feelings of being worthless, useless and dismissed since they lost a lot as a result of their CKD:

*‘I used to be [name] from my job, then from the horses and now … now I’m [name] from nothing. I’m nothing’! (R41, F, 46–55 years, GFR 20)*

They further expressed how the disease changed their attitude/way of behaving in contacts with others. In some cases, this gave rise to feelings of insecurity, such as ‘does the other person like the new me’?:

*‘I sometimes wonder: do they still like me? They know me as the one dancing on the tables. I’m now the one who is sitting in a corner, chatting and drinking somewhat. People are going to see another part of you that is getting bigger and bigger than the part of you they used to know’. (R6, F, 26–35 years, GFR 45)*

#### Education, work and social security

Many participants were no longer able to work or were only able to work part-time, which could lead to financial pressure. Fatigue was the most common reason for this:

*‘I was working full-time; it was too heavy. I fell asleep during my work! Really embarrassing’! (R5, F, 26–35 years, GFR 20)*

Making the choice to stop working or to decrease the number of hours was experienced as a tough decision that led to various feelings, such as the feeling of losing everything and being put aside to feelings of being relieved or feeling empty. Work was often described as important since it gave people a sense of identity. As a result, sometimes participants tried to do their best to keep working. Maintaining a job was also important since people did not want to receive alimony or have financial problems. Receiving alimony was associated with financial problems and with embarrassment and fear of prejudice:

*‘It is a negative label that is put on your forehead’. (R41, F, 36–45 years, GFR 20)*

Participants abandoned other activities and social contacts just so that they could work as much as possible. They became exhausted and did not have sufficient energy for other activities, which led to the question: ‘do I want this?’:

*‘I really want to continue working but I don’t have the energy for other things. I’m not sure anymore if work is worth all the sacrifices’. (R27, F, 46–55 years, GFR 38)*

Workplace adaptations were sometimes possible, but led to less satisfaction or a sense of loss of status. Participants also had to deal with the reactions of their employer and company doctor. Some participants experienced a lot of understanding whereas others did not experience much empathy. In addition, company doctors and those conducting medical examinations for social security often know little about the impact of CKD in earlier stages:

*‘The doctor who had to decide whether I was able to work or not said: “come back if you have to start with dialysis; for now we are not going to change anything”.’ (R23, F, 36–45 years, GFR 25)*

Participants further, expressed feelings of being punished for being sick.

*‘Did I choose to have this disease? I don’t think so … but it feels like being punished’. (R8, F, 36–45 years, GFR 23)*

### Healthcare

#### Education

Participants experienced the care received as being insufficient and not attuned to their needs and situation. One problem is the lack of information after diagnosis and during progression of the disease. Most participants were shocked by the diagnosis, even if they knew there was a high possibility of having the disease. Realising that the disease is progressive and unpredictable was difficult and participants needed some time to accept the diagnosis. They desired more information about their disease and its consequences, but this was often lacking. The information they received did not fit their situation since it was focused on (pre)dialysis and transplantation:

*‘The information does not fit. It’s like you fall between two stools a little’. (R28, M, 26–35 years, GFR 39)*

#### Contact with professionals

Patients expressed various experiences with their nephrologists. Some were really satisfied whereas others were unsatisfied. Dissatisfaction was often the result of a lack of personal contact and being taken seriously:

*‘I want to be more than my renal function. They don’t see you as a person’. (R2, F, 46–55 years, GFR 27)*

*‘The nephrologist is just looking at you when you explain your fatigue. And the only thing he does, is telling you: “It doesn’t fit with your GFR: it doesn’t fit”.’ (R6, F, 26–35 years, GFR 45)*

#### Multidisciplinary care and self-management

Participants lacked multidisciplinary care, for instance from a social worker or dietician other than their physician. They also lacked self-management opportunities:

*‘I knew I had to follow a diet, but nobody has ever told me that and nobody has ever supported me starting a diet’. (R2, F, 46–55 years, GFR 27)*

Participants who received such care were more satisfied, but the offer often came too late so patients had to arrange their care themselves, wondering where to find adequate and valid care:

*‘They offered me such care after years. It was too late, I had already solved my problems on my own’. (R8, F, 36–45 years, GFR 23)*

#### Experimental care and future treatment

Because of a lack of treatment options experienced, some participants took part in experimental treatments/research. By participating in such projects they hoped to slow the progression of their disease. However, being the subject of scientific research sometimes resulted in a more frequent confrontation with the disease:

*‘The prospects of the medication were promising. Yes, I jumped for joy when I could participate. It’s exciting: you just intensely hope you may profit yourself. But there is also another side of the medal. Every 3 months you are being checked and informed about your blood values. A first injection, a second injection, a subsequent test, yet another test. You just feel like a patient and that’s not always pleasant’. (R20, F, 36–45 years, GFR 42)*

Some participants were also already thinking about future therapies. They did not always feel supported by professionals who did not want to give information about future treatments or professionals who advised patients not to worry about the future:

*‘The nephrologist advised me not to think about dialysis or transplantation yet because I’m not in that stage of the disease yet. But I know I will need it 1 day so it’s not that easy to put all those emotions and doubts away’. (R20, F, 36–45 years, GFR 42)*

Other opposite problems arose when pre-emptive transplantation was already mentioned as a possibility. Professionals, according to patients, talked too easily about finding a donor.

*‘The physician said to me: “You can avoid dialysis if you are able to find a donor”. I was looking at my family and friends and then decided: I’m not going to ask them! I don’t want to take the risk. What if something happens to them’! (R5, F, 26–35 years, GFR 20)*

Talking about pre-emptive transplantation was experienced as difficult. Participants did not know how to introduce the subject or they felt pressurised to find a donor. Some participants who did ask their relatives to become a donor had to deal with negative reactions. Participants also found it difficult to wait for a treatment, such as transplantation, to be carried out.

### Psychological aspects

#### Deterioration - insecurity and trust

Patients with CKD experienced difficulties that they had to deal with. One difficulty was dealing with a (slow) decrease of kidney function and insecurity about the future:

*‘You are not quite sure about the possibilities of your body, now and in the future. It is such a silent disease. It may become worse without you noticing it’. (R27, F, 46–55 years, GFR 38)*

#### Coping

Participants used many strategies to deal with their disease and restrictions. Most of the time they were able to deal with the disease and its consequence and they showed a considerable amount of resilience. Trying to stay in control was one of the mentioned strategies. A way of doing this was by following a diet or initiating other lifestyle adaptations:

*‘I have a diet and this makes me happy since it gives me a sense of control: I can do something to stop the disease’. (R27, F, 46–55 years, GFR 38)*

Having no possibility to control the disease was associated with stress, whereas lifestyle adaptations provided a sense of control:

*‘There is not much I can do to stop it. My blood pressure is good and a diet doesn’t help. Living a healthy life is the only thing. This can be frustrating sometimes. Something happens in your body and there is nothing you can do, nothing but wait and see’. (R26, M, 18–25 years, GFR 40)*

Some participants however did not want to adapt their lifestyle because they expected these changes to negatively impact their quality of life:

‘*My daughter is pushing me towards a diet. No way! It is not going to happen since my quality of life is more important than quantity’! (R18, M, 56–65 years, GFR 39)*

Another way to keep a sense of control was by setting boundaries and anticipating bodily signals. Finding and maintaining personal boundaries were reported as time- and energy consuming but helpful. Participants experienced additional barriers because of the incomprehension of the environment and deterioration of the disease:

*‘The environment doesn’t know your possibilities and limitations. They are asking you … can you please do this and that … And my reply: “Of course … I will do it all”.’ (R22, F, 36–45 years, GFR 35)*

Other coping strategies mentioned included focussing on the present and possibilities instead of on the insecure future:

‘*I try to live day to day and see what I am able to do right now. I tend to ignore the future a little. Thinking about that is too overwhelming and emotional, so I just try not to do that’. (R20, F, 36–45 years, GFR42)*

This could be difficult, but it was seen as being just as important as staying positive and hopeful:

*‘The notion that it will be more difficult in the future is always there. I may not have many problems right now, but the sword of Damocles is always hanging over my head’. (R26, M, 18–25 years, GFR 40)*

Humour also helped to place problems in perspective.

Communication and openness about restrictions were also mentioned as important. Openness led to understanding, but it was daunting because people feared the possible negative consequences of being open:

*‘I find that difficult, being dependent or being stigmatised. I just wanted to take part as long as possible. Especially in the past few years, I really crossed my limits … because I did not want to be seen as weak’. (R4, F, 56–65 years, GFR 23)*

Energy management was a final way to deal with restrictions. Patients tried to use their diminished energy as effectively as possible. Making choices was reported as being necessary but also difficult:

*‘I sometimes sacrifice things—cycling, swimming, yoga—if something else is more important to me. There are no “must-dos” anymore’. (R4, F, 56–65 years, GFR 23)*

*‘These are difficult choices. What is most important to me? Work or being able to do something in the evenings and during the weekend’? (R26, M, 18–25 years, GFR 40)*

In the end, acceptance was needed but participants needed time to reach a state of acceptance, and sometimes acceptance was impossible for patients:

*‘It is funny though, because I went from a state of denial to total acceptance, I believe’. (R6, F, 26–35 years, GFR 45)*

*‘You just get it served and have to eat it like that, whether you like it or not’. (R33, F, 56–65 years, GFR 38)*

## Discussion

The existing literature gives us insight into the QoL of patients with CKD. Their QoL seemed to be decreased in all stages of the disease [3, 4] and it is unclear whether or not the GFR influences the QoL [3, 4].

This study gives further insight into the patients’ experiences of living with moderate-to- severe CKD. Participants in this study mentioned many experiences that had not previously been described in the literature and are not yet sufficiently recognised in daily practice.

This study shows, in general, that living with decreased renal function already influences the QoL of patients across several domains. The disease/decreased renal function has physical, social, societal and psychological impacts and seems to influence the QoL in several ways.

Four main findings were mentioned most often and were emphasised the most by participants. We will discuss these findings in more detail below.

The first main finding is the fact that 75 % of the participants who filled out the CIS questionnaire (*n* = 32) reported severe fatigue on the CIS questionnaire. Fatigue was also mentioned very often in the interviews and focus groups. Fatigue is often described in the literature as being very problematic in CKD, especially during dialysis [19–24]. The prevalence of (severe) fatigue in dialysis patients ranges from 60 to 97 % [22]. Fatigue after transplantation has also already been described [25, 26]. Dutch patients who had received a kidney transplant were significantly more often severely fatigued (39 %) compared to matched controls [26].

However, prior to this study, fatigue was not mentioned in combination with a GFR of between 30 and 45, while this study suggests that fatigue can be seen as a significant problem for the majority of patients with moderate-to-severe CKD. Patients do not feel taken seriously by physicians who ignore the fatigue or tend to allocate it to causes other than renal disease. Problems are only expected if the GFR drops below 30, as was also described in Dutch and international education materials for patients. The results of this study put forth the need for more research on fatigue in this stage of the disease, more attention for fatigue in earlier stages of the renal disease and the possible adaptation of education materials. Attention should also be given to the strategies already used by patients in order to deal with their fatigue. Professionals should focus their advice on the strategies of patients in order to strengthen them.

The second related finding is that participants really struggled with the lack of acknowledgement of their complaints. This struggle is partly caused by the patients’ own thoughts and assumptions, the invisibility of the disease and societal norms of economic productivity and independency. It is also caused by the education and attitude of professionals (‘you cannot have these complaints right now; you are only allowed to have complaints with GFR lower than 20 or 30’). These factors led to the finding that these patients are desperately searching for acknowledgement. If they receive acknowledgement, they can be open about their complaints. If they do not receive acknowledgment, the patient may choose to live a life full of secrecy. They do not want to be open, because many have lost jobs, income and economic self-sufficiency due to (openness about) CKD or they are afraid this will happen.

Searching for acknowledgment is a well-known theme in the (illness) experiences of patients with a chronic disease. Often, the moment of getting the diagnosis or the diagnosis itself gives a certain form of acknowledgement. The diagnosis of itself may, provide exemption from role obligations, whereby people no longer feel blamed for their disability [27]. In the case of moderate CKD, this seems to work the other way around: the diagnosis in itself does not give enough clarification and justification of the complaints, which results in a search for acknowledgment.

Acknowledgment of the complaints of patients by professionals and changing education towards patients are the first step to changing the image formation and acceptance of this group of patients with these specific complaints.

The third main finding concerns the problems participants already experience regarding their participation in work. Nowadays, attention is mostly given to work problems as experienced by patients in the pre-dialysis stage (stage 4) or during dialysis (stage 5). The literature shows that people on dialysis have problems combining their treatment with a paid job; participation rates of around 24 % in dialysis patients aged below 65 are described [28–30]. No differences in participation in life activities (physical function, travel, recreation, freedom and work) were found between patients receiving haemodialysis or peritoneal dialysis [31]. Pre-dialysis patients (stage 4) also already experience work-related problems. A Dutch study showed that only 35 % of the patients, aged 18–64 years, had a paid job at the start of the treatment [32]. In addition to problems completing paid work, around 30 % of the pre-dialysis patients (stage 4) and more than 50 % of the dialysis patients (stage 5) reported stressors with respect to work [33].

The literature does not give insight into possible participation problems as experienced by patients in stage 3. Our study shows that patients with less severe renal failure (GFR between 30 and 45, stage 3), already experience problems with continuing their work as a result of experienced fatigue. They have to make tough decisions regarding their work, which causes a lot of emotions. This study shows, in line with social research, that losing your job does not only lead to a loss of economic self-sufficiency and the loss of income, but it also leads to a loss of identity [34]. This loss of identity or loss of self makes it difficult to accept the diagnosis and integrate it into the patients’ lives [35, 36]. This loss of identity and other losses can be a difficult process and patients in our study were keen on avoiding this situation of loss of work and possibly their identity by trying to overcompensate and by pushing themselves to the limit. It seems important to give more attention to the work problems people with moderate CKD (stage 3) may experience, especially because of the significant impact on identity [34] and the influence of income on QoL [4]. Attention should be given to the problems as experienced in order to prevent further problems and the possibility of increasing loss of participation.

The final main finding is the wish to have control. Participants wanted to do something to stop or slow the progression of the disease and most of the time, they show considerable amounts of resilience. Not being able to do something created a sense of loss of control and influenced the experienced resilience. The focus on doing the things you can in order to prevent further problems is in line with the underlying ideas of the Chronic Care Model and self-management. The Chronic Care Model stresses the importance of proactive care focused on supporting people’s ability to self-manage their health [37]. The model advocates an active role for patients, who are encouraged to become more knowledgeable about factors affecting their condition(s) and become more actively involved in decisions about their care [37].

Self-management has also gained more and more attention in the care of patients with CKD/ESRD. Most programmes are, however, aimed at the pre-dialysis and dialysis stages (e.g. [38]), rather than at this stage. Participants were very dissatisfied with the fact that they did not receive advice on how to change their lifestyle. Advice is only suggested and supported at the pre-dialysis stage. Participants received this too late.

## Conclusions

This study gives new insights into an expanding group of patients (http://www.ekha.eu) with CKD that has not yet attracted much research attention. Information about this group has not yet been described in the literature.

Critical readers may mention that this study is ‘just’ a qualitative study, which makes the results difficult to generalise. The sample size was indeed relatively small compared with that of a quantitative study, but it was very reasonable for a qualitative one. The results can be seen as valid since we reached saturation [39, 40]. Nevertheless, the readers must decide whether the results are recognisable and applicable for their practice and which results can be used in their own practice of working with patients with CKD. Future quantitative research can help us to further validate the findings of this study.

Meanwhile, this study gives many rich insights into the experiences and life-world of patients with a moderate-to-severe renal function. Such insights were lacking and are needed to improve healthcare and the QoL of this group of patients. Ideally, professionals should be more aware of the problems patients already may experience in earlier stages of the disease and how these problems may influence QoL. They should ask patients about possible problems and they should acknowledge and not devaluate those problems, giving patients the support they need in order to solve problems as much as possible. Giving attention to the problems experienced by patients and trying to solve those problems is in line with a relative new trend in healthcare: personalised care planning.

Personalised care planning is a collaborative pro-active process used in the management of chronic diseases in which patients and professionals identify and discuss problems caused by or related to the patient’s condition, developing a plan for tackling these. In essence, it is a conversation or series of conversations, in which they jointly agree on goals and actions for managing the patient’s condition [37]. Personalised care planning aims at ensuring that the individuals’ values and concerns that shape the way long-term conditions are managed.

Instead of focusing on a standard set of disease management processes determined by healthcare professionals, this approach encourages patients to select treatment goals and work with clinicians to determine their specific needs for treatment and support [37]. The results from this study can be used to set goals. The results also pinpoint the importance of holistic care, as Mangin previously described. Ideally the care for people with multiple long-term conditions like CKD should be holistic, which means person-centred rather than disease-focused. It should be responsive to individuals’ experiences of illness and treatment effects and persons’ individual priorities [37]. Personalised care planning leads to people’s increased capability to self-manage their condition when compared to usual care, as described in a recent systematic review [37].

### Ethics and consent to participate

The Medical Ethics Committee of the VU Medical Center approved this study and the procedures followed were in accordance with the ethical standards of the Medical Ethics Committee of the VU Medical Center. All participants voluntarily took part and none of them declined our request to participate. All participants gave verbal informed consent.

### Consent to publish

All participants gave consent for using their interviews anonymously.

### Availability of data and materials

Extra information can be found in the supplementary files.

## Appendix 1

### Topiclist interviews

Introduction

- Introduction interviewers
- Background and objectives research
- Audio recording: explanation goal and getting consent?
- Privacy and use of data and right to stop the interview or to skip questions

Background participant

- Actual daily activities: (voluntary) work, hobbies, et cetera
- Work, education (history)
- Family setting/Matrimony

Illness history

- Diagnosis (including heredity)
- Actual treatment and treatment history
- Co-morbidity?
- Actual health status (GFR, complaints)

Eventual influence of disease on quality of life

- Daily activities (ADL and housekeeping)
- Social contacts and intimate relationships (including sexuality)
- Childwish and family life
- Hobbies and sport activities
- (Voluntary) work (including UWV, et cetera)
- Financial aspects
- Physical functioning

Care and support

- Actual received care and care history
- Experiences with care
- Needs and wishes regarding care and support
- Possible suggestions for improvement of care/support
- Possible suggestions for support by patient organisation

Coping

Closing remarks/evaluation interview/any further support needed?

## Appendix 2

### Protocol focus groups

*Program*

1. Welcome, goals of the study and today (validation of results and formulation of recommendations), consent and consent for audio recording, privacy
2. Filling in the CIS-fatigue
3. Getting to know each other
4. Validation of results: participants receive an envelope with the themes as found in the interviews. Participants are asked to attach each theme on the red or green piece of paper on the wall. If they recognize the theme, they attach it on the green paper and if they do not recognize the theme, they attach it on the red paper. Themes that are missed have to be written down on the white paper.
5. Dialogue about step 3: finding similarities and differences and pinpointing the most important themes.
6. Formulation of recommendations based on the findings.
7. Conclusions, evaluation closing remarks (including giving of small attention for participants and explain how to declare the travel costs) and check needs of participants after this session/emotional impact (f.i. do we need to call tomorrow)*.*

## Abbreviations

CKD: chronic kidney disease
ESRD: end-stage renal disease
GFR: glomerular filtration rate
HRQOL: health related quality of life
NVN: Dutch Kidney Patient Association
QoL: quality of life

## References

[1] Jha V, Garcia-Garcia G, Iseki K (2013). Chronic kidney disease: global dimension and perspectives. Lancet.

[2] White SL, Chadban SJ, Jan S, Chapman JR, Cass A (2008). How can we achieve global equity in provision of renal replacement therapy?. Bull World Health Organ.

[3] Hajeong L, Yun Jung O, Myounghee K (2012). The association of moderate renal dysfunction with impaired preference-based health-related quality of life: 3^rd^ Korean national health and nutritional examination survey. BMC Nephrol.

[4] Foresti Lemos C, Palmeira Rodrigues M, Veiga JRP (2015). Family income is associated with quality of life in patients with chronic kidney disease in the pre-dialysis phase: a cross sectional study. Health Qual Life Outcomes.

[5] Kuper A, Reeves S, Levinson W (2008). Qualitative research. An introduction to reading and appraising qualitative research. Br Med J.

[6] Meadows ML, Morse JM, Morse M, Swanson JM, Kuzel AJ (2001). Constructing evidence within the qualitative project. The nature of qualitative evidence.

[7] Abma TA, Broerse J (2010). Patient participation as dialogue: setting research agendas. Health Expect.

[8] Schipper K, Abma TA, van Zadelhoff E (2010). What does it mean to be a patient research partner? An ethnodrama. Qual Inq.

[9] Hewlett S, de Wit M, Richards P (2006). Patients and professionals as research partners: challenges, practicalities, and benefits. Arthritis Rheum.

[10] De Wit M (2013). Patient participation in rheumatology research. A four level responsive evaluation.

[11] Nierse C, Schipper K, van de Griendt J, Abma TA. Collaboration and co-ownership in research. Dynamics and dialogues between patient research partners and professional researchers in a research team. Health Expect. 2011. doi: 10.1111/j.1369-7625.2011.00661. 10.1111/j.1369-7625.2011.00661.x PMC5060620 21332617

[12] Oliver M (1992). Changing the social relations of research production. Disabil Handicap Soc.

[13] Schipper K (2012). Patient participation and knowledge.

[14] Rubin HJ, Rubin IS (2012). Qualitative interviewing: the art of hearing data.

[15] Braun V, Clarke V (2006). Using thematic analysis in psychology. Qual Res Psychol.

[16] Mays N, Pope C (1995). Qualitative research: rigour and qualitative research. Br Med J.

[17] Meadows LM, Morse JM, Morse JM, Swanson JM, Kuzel AJ (2001). Constructing evidence within the qualitative project. The nature of qualitative evidence.

[18] Vercoulen JH, Swanink CM, Fennis JF (1994). Dimensional assessment of chronic fatigue syndrome. J Psychosom Res.

[19] Karakan S, Sezer S, Ozdemir FN (2011). Factors related to fatigue and subgroups of fatigue in patients with end-stage renal disease. Clin Nephrol.

[20] Bossola M, Vulpio C, Tazza L (2011). Fatigue in chronic dialysis patients. Semin Dial.

[21] Jhamb M, Weisbord SD, Steel JL, Unruh M (2008). Fatigue in patients receiving maintenance dialysis: a review of definitions, measures, and contributing factors. Am J Kidney Dis.

[22] Murtagh FE, Addington-Hall J, Higginson IJ (2007). The prevalence of symptoms in end-stage renal disease: a systematic review. Adv Chronic Kidney Dis.

[23] Weisbord SD, Fried LF, Arnold RM (2005). Prevalence, severity, and importance of physical and emotional symptoms in chronic hemodialysis patients. J Am Soc Nephrol.

[24] Lee BO, Lin CC, Chaboyer W, Chiang CL, Hung CC (2007). The fatigue experience of haemodialysis patients in Taiwan. J Clin Nurs.

[25] Goedendorp MM, Hoitsma AJ, Bloot L (2013). Severe fatigue after kidney transplantation: a highly prevalent, disabling and multifactorial symptom. Transpl Int.

[26] Schipper K, Abma TA, Koops C (2013). Sweet and sour after renal transplantation: a qualitative study about positive and negative consequences of transplantation. Br J Health Psychol.

[27] Scambler G (2009). Health-related stigma. Sociol Health Illn.

[28] Braun Curtin R, Oberly ET, Sacksteder P, Friedman A (1996). Differences between employed and non-employed dialysis patients. Am J Kidney Dis.

[29] Jansen DL, Rijken M, Heijmans M, Boeschoten EW (2010). Perceived autonomy and self-esteem in Dutch dialysis patients: the importance of illness and treatment perceptions. Psychol Health.

[30] Theorell T, Konarski-Svensson JK, Ahlmen J, Perski A (1991). The role of paid work in Swedish chronic dialysis patients - a nation-wide survey: paid work and dialysis. J Intern Med.

[31] Purnell TS, Auguste P, Crwes DC (2013). Comparison of life participation activities among adults treated by hemodialysis, peritoneal dialysis, and kidney transplantation: a systematic review. Am J Kidney Dis.

[32] Van Manen JG, Korevaar JC, Dekker FW, Reuselaars MC, Boeschoten EW, Krediet RT (2001). Changes in employment status in end-stage renal disease patients during the first year of dialysis. Perit Dial Int.

[33] Ekelund ML, Andersson SI (2007). Elucidating issues stressful for patients in predialysis and dialysis: From symptom to context. J Health Psychol.

[34] Stryker S, Burke PJ (2000). The past, present and future of an identity theory. Soc Psychol Q.

[35] Nätterlund B, Sjöden P, Ahlström G (2001). The illness experience of adult persons with muscular dystrophy. Disabil Rehabil.

[36] Charmaz K (1983). Loss of self: a fundamental form of suffering in the chronically ill. Sociol Health Illn.

[37] Coulter A, Entwistle VA, Eccles A, Ryan S, Shepperd S, Perera R (2015). Personalised care planning for adults with chronic or long-term health conditions. Cochrane Database Syst Rev.

[38] Chen SH, Tsai YF, Sun CY (2011). The impact of self-management support on the progression of chronic kidney disease--a prospective randomized controlled trial. Nephrol Dial Transplant.

[39] Barbour RS (2001). Checklists for improving rigour in qualitative research: A case of the tail wagging the dog?. Br Med J.

[40] Mays N, Pope C (2000). Qualitative research in health care. Assessing quality in qualitative research. Br Med J.

